# Tanshinone I improves renal fibrosis by promoting gluconeogenesis through upregulation of peroxisome proliferator-activated receptor-γ coactivator 1α

**DOI:** 10.1080/0886022X.2024.2433710

**Published:** 2024-12-08

**Authors:** Yanfang Bai, Hui Wen, Junyan Lin, Xinying Liu, Hua Yu, Ming Wu, Ling Wang, Dongping Chen

**Affiliations:** a Department of Nephrology, Shuguang Hospital Affiliated to Shanghai University of Traditional Chinese Medicine, Shanghai, China; b TCM Institute of Kidney Disease of Shanghai University of Traditional Chinese Medicine, Shanghai, China; c Key Laboratory of Liver and Kidney Diseases, Ministry of Education, Shanghai Key Laboratory of Traditional Chinese Clinical Medicine, Shanghai, China; d The Seventh People’s Hospital, Shanghai University of Traditional Chinese Medicine, Shanghai, China; e Department of Rheumatology, Shanghai Tenth People’s Hospital of Tongji University, Shanghai, China; f Shanghai Zhabei District Central Hospital, Shanghai, China; g Department of Nephrology, Shanghai Tenth People’s Hospital of Tongji University, Shanghai, China

**Keywords:** Tanshinone I, gluconeogenesis, PGC1α, renal fibrosis, chronic kidney disease

## Abstract

**Background:**

Renal fibrosis, a hallmark of chronic kidney disease, is closely associated with dysregulated gluconeogenesis. Tanshinone I (Tan I), a bioactive compound derived from the traditional Chinese medicine Danshen, exhibits antifibrotic and anti-inflammatory properties. However, its effects on gluconeogenesis and the mechanisms through which it alleviates renal fibrosis remain unclear. This study aimed to investigate whether Tan I promotes gluconeogenesis and mitigates renal fibrosis.

**Methods:**

Both *in vivo* and *in vitro* experiments were conducted. A unilateral ureteral obstruction (UUO) mouse model was used. Masson’s trichrome, HE, and immunofluorescence staining, along with Western blotting, were employed. Lactate concentrations and a pyruvate tolerance test were conducted to assess glucose metabolism. *In vitro*, HK2 cells and primary renal tubular cells were treated with transforming growth factor-β (TGFβ) to induce fibrosis, and the effects of Tan I on glucose and lactate levels were examined.

**Results:**

In the UUO model, Tan I reduced fibrosis, decreased lactate accumulation, and modulated fibrosis markers while upregulating gluconeogenesis markers. Tanshinone I restored impaired renal gluconeogenesis, as evidenced by increased pyruvate levels. *In vitro*, Tan I inhibited fibrosis, reduced lactate levels, and increased glucose levels in cell supernatants. It also restored gluconeogenesis protein expression and decreased fibrotic protein levels. Peroxisome proliferator-activated receptor-γ coactivator (PGC1α) expression was downregulated in UUO and TGFβ-stimulated models, and Tan I reversed this downregulation. Inhibition of PGC1α in TGFβ-stimulated cells counteracted the antifibrotic and gluconeogenesis-promoting effects of Tan I.

**Conclusions:**

Tanshinone I ameliorated renal fibrosis by enhancing gluconeogenesis through upregulation of PGC1α.

## Introduction

In recent years, the morbidity and mortality rates of chronic kidney disease (CKD) have been rising annually, making it a significant threat to human health and public safety [1–3]. Renal fibrosis, a key pathological feature of CKD, is characterized primarily by excessive deposition of extracellular matrix, activation of renal interstitial fibroblasts, and renal epithelial–mesenchymal transition [4]. This condition results in the destruction of renal structure and loss of function, ultimately leading to death. The primary pathological mechanisms of renal fibrosis include inflammatory responses, oxidative stress, apoptosis, and abnormal cellular metabolism [5]. Although some treatments can slow disease progression, effective therapies remain limited, highlighting the need for new therapeutic strategies. Traditional Chinese medicine (TCM) shows promise in the treatment of renal fibrosis, but its mechanisms of action remain unclear.

Gluconeogenesis is a series of enzymatic reactions in which non-carbohydrate substrates (e.g., lactic acid, glycerol, and amino acids) are converted into glucose or glycogen, primarily in the liver and kidneys [6,7]. This process is essential for maintaining blood glucose levels and cellular energy metabolism. Recent studies have increasingly focused on the role of gluconeogenesis in renal diseases. Metabolic dysregulation is considered a crucial factor in the progression of renal fibrosis. Excessive deposition of extracellular matrix and inflammatory responses elevate the kidney’s energy demand, while diminished gluconeogenesis results in insufficient energy supply, exacerbating cellular stress and injury, and thereby accelerating the fibrotic process [8–10]. It has been shown that proximal tubule cells are able to activate surrounding mesenchymal fibroblasts by secreting pro-fibrotic factors, such as transforming growth factor-β (TGF-β) and CTGF, prompting them to differentiate into myofibroblasts, thereby promoting the progression of fibrosis [11–13]. Regulating gluconeogenesis may therefore be pivotal in improving the kidney’s metabolic environment and mitigating the fibrotic process.

Peroxisome proliferator-activated receptor-γ coactivator 1α (PGC1α) is a key metabolic regulator involved in various metabolic processes, including gluconeogenesis [14], lipid oxidation [15], and mitochondrial biogenesis [16]. PGC1α regulates the expression of metabolic genes by interacting with various nuclear receptors (e.g., PPARs and ERRs) to maintain cellular energy balance and metabolic homeostasis. In the kidney, PGC1α expression and function are crucial for maintaining normal metabolism in renal tubular cells. PGC1α plays a protective role in renal diseases, attenuating the progression of renal fibrosis by enhancing mitochondrial function, reducing oxidative stress, and inhibiting inflammatory responses [17]. Consequently, PGC1α has become an important target for studying renal fibrosis.

Tanshinone I (Tan I) is a major active ingredient extracted from the TCM Danshen (*Salvia miltiorrhiza*) and exhibits various biological activities, including anti-inflammatory, antioxidant, and antifibrotic effects [18]. Tanshinones and their compounds have demonstrated significant therapeutic effects in various disease models. They can attenuate fibrosis in tissues such as the liver, kidney, lung, and heart through multiple pathways [19–22]. Tanshinone I also has notable nephroprotective effects, promoting the metabolism of aristolochic acid and mitigating aristolochic acid nephropathy (AAN), thereby exerting a protective effect on the kidneys [23]. Our previous study showed that Tan I can improve renal fibrosis [24], but the mechanism of action remains unclear.

Based on this research background, the present study aimed to investigate whether Tan I promotes gluconeogenesis through upregulation of PGC1α, thereby ameliorating renal fibrosis.

## Materials and methods

### Animal experiments

The animal experiments in this study were approved by the Ethics Committee for Animal Experiments of Shanghai University of Traditional Chinese Medicine. C57BL6/J male mice, weighing between 20 and 22 g, 8 weeks of age and provided by Shanghai Model Organisms Center Inc. (Shanghai, China), were used. All mice were SPF-grade and housed in the Animal Experiment Center of Shanghai University of Traditional Chinese Medicine (Shanghai, China), managed in accordance with local regulations and guidelines.

For left unilateral ureteral obstruction (UUO), mice were anesthetized intraperitoneally with 0.8% sodium pentobarbital (8 mg/kg) (Sigma, St. Louis, MO, CAS: 57-33-0). During surgery, an incision was made on the left side of the abdomen to expose the left ureter, which was then ligated and sutured. Mice in the sham-operated group underwent the same surgical procedure without ureteral ligation. A total of 24 mice were used and randomly divided into four groups of six mice each: (1) Sham/DMSO group; (2) Sham/Tan I group; (3) UUO/DMSO group; (4) UUO/Tan I group. Starting on the first day after surgery, mice were injected intraperitoneally with DMSO or 50 mg/kg Tan I (Topscience, T2907, Shanghai, China) daily for 14 days. After 14 days, mice were anesthetized with sodium pentobarbital, and kidney tissues were collected for subsequent analysis. 0.8% sodium pentobarbital was injected intraperitoneally and taken under anesthesia, and euthanasia by spinal cord subluxation in mice.

### Mouse primary cell extraction

*2 mg/mL type II collagenase digestion working solution*: Prepare the incubation solution by formulating trypsin inhibitor (Beyotime, Cat: SG2033, Shanghai, China), DNase I (Beyotime, Cat: D7073, Shanghai, China), and Krebs-Ringer buffer (Solarbio, Cat: G0430, Beijing, China). Dissolve collagenase type II (Worthington, LS004174, Columbus, OH) in serum-free DMEM/F12 medium, filter to remove bacteria, and obtain a 10 mg/mL collagenase type II solution. Then, mix with the incubation solution to prepare a 2 mg/mL type II collagenase digestion working solution.

For primary renal tubular cell extraction, C57BL6 mice, approximately 4 weeks old, were used. Mice were anesthetized with an intraperitoneal injection of 0.8% sodium pentobarbital (8 mg/kg). Kidneys were isolated and placed in cold PBS. On an ultra-clean bench, the renal hilum and peritoneum were removed, and the medullary part of the kidney was isolated. The cortical part was cut into 4–5 slices with a scalpel blade. Add 1 mL of the 2 mg/mL type II collagenase digestion working solution, and incubate with shaking at 37 °C for 5 min. Remove the supernatant, and add 1 mL of the 2 mg/mL type II collagenase digestion working solution to the remaining renal tissues. Incubate with shaking at 37 °C for 45 min. After digestion, add 1 mL of DMEM/F12 medium containing 10% fetal bovine serum (FBS), 0.5% penicillin/streptomycin, and ITS. Centrifuge at 800 rpm for 5 min, remove the supernatant, and add 1 mL of DMEM/F12 medium containing 10% FBS, 0.5% penicillin/streptomycin, and ITS. Centrifuge again, remove the supernatant, and resuspend the pellet in 1 mL of 10% FBS/DMEM/F12 medium. Transfer to a large dish containing 10% FBS/DMEM/F12 medium for primary cell culture.

### Cell culture and intervention

Human kidney 2 (HK2) cells, a human renal proximal tubular epithelial cell line, were obtained from the Cell Bank of Shanghai Institute of Biological Sciences, Chinese Academy of Sciences (Shanghai, China). HK2 cells were cultured in DMEM/F12 medium containing 10% FBS and 0.5% penicillin/streptomycin at 37 °C and 5% CO_2_. Cells were inoculated into a six-well plate, and when the cell density reached approximately 50%, the medium was changed to one containing 0.5% FBS to starve the cells for about 8 h. After starvation, the medium was replaced with fresh DMEM/F12 containing 0.5% FBS and 2.5 ng/mL TGFβ (100-21, Peprotech, Rocky Hill, NJ), with either control solution or Tan I added. After 48 h of treatment, the supernatant and cellular proteins were collected for subsequent analysis.

Primary renal tubular cells, extracted directly from mice, were cultured in DMEM/F12 medium containing 10% FBS, 0.5% penicillin/streptomycin, and ITS. The cells were incubated in a 5% CO_2_ incubator at 37 °C. Primary renal tubular cells were inoculated in six-well plates, and when the cell density reached approximately 80%, the medium was replaced with 2 mL of medium containing 0.5% FBS for modeling intervention. Cells were stimulated with 10 ng/mL TGFβ and treated with either control solution or Tan I. After 48 h of treatment, the supernatant and cellular proteins were collected for subsequent analysis.

### Use of PGC1α inhibitor

Primary renal tubular cells were inoculated in six-well plates, and modeling intervention was performed when the cell density reached approximately 80%. The experiment was divided into three groups: (1) TGFβ group: stimulated with 10 ng/mL TGFβ added to the culture medium; (2) TGFβ + Tan I group: Tan I was added in addition to TGFβ stimulation; (3) TGFβ + Tan I + PGC1α inhibitor group: a PGC1α inhibitor (20 mM) was added alongside TGFβ and Tan I interventions. After 48 h of treatment, the supernatant and cellular proteins were collected. The expression of fibrosis and gluconeogenesis-related proteins was assessed by Western blotting (WB) and other methods.

### Mouse glucose tolerance experiments

A pyruvate tolerance test (PTT) was conducted on day 9 following UUO surgery. Pyruvate, an important substrate for gluconeogenesis, was used to assess the gluconeogenic capacity of mice by monitoring the rise in blood glucose levels after intraperitoneal injection of sodium pyruvate solution. Fasting began at 4 pm on the day before the experiment and continued for 16 h. Adequate water was provided during the fasting period to maintain the normal physiological state of the mice. The experiment commenced at 8 am on the second day. Basal fasting blood glucose levels were measured using a glucometer and blood glucose test strips. Sodium pyruvate (A100342, Shanghai Shenggong Technology, Shanghai, China) was diluted in PBS, and a 2 g/kg sodium pyruvate solution was administered intraperitoneally. Blood glucose levels were measured and recorded at 10, 20, 30, 60, 90, and 120 min post-injection. At the end of the experiment, mice were provided with chow. Changes in blood glucose values at each time point were analyzed to evaluate the gluconeogenic capacity of the mice.

### Gluconeogenic intermediate measurement

The renal cortex was collected for protein extraction. Protein samples were extracted using 10 times normal saline through mechanical disruption. Lactate content was measured with a lactate test kit (A019-2-1, Nanjing Jiancheng Bioengineering Institute, Nanjing, China) by determining the OD value at 530 nm. All measurements were performed according to the manufacturer’s instructions and were standardized by protein concentration.

### Masson’s trichrome and HE staining

Kidneys were fixed in 4% paraformaldehyde and embedded in paraffin. Samples were sectioned into 4–5 μm slices using a paraffin slicer and mounted on slides. HE staining and Masson’s trichrome staining were performed subsequently. Images were obtained using a microscope (Nikon 80i, Nikon, Tokyo, Japan) to observe renal morphology and interstitial collagen fibers. Five fields of view per section were selected in a clockwise direction. The area of Masson’s trichrome-stained positive renal tubular interstitial collagen fibers in each sample was quantified using ImageJ software (Media Cybernetics, Rockville, MD).

### Immunohistochemistry (IHC)

Kidney tissues from Sham, UUO, and Tan I groups were fixed in 4% paraformaldehyde, dehydrated and cut into 5 µm thick sections. Paraffin sections were deparaffinized to water, antigenically repaired, endogenous peroxidase blocked, BSA blocked, primary antibody overnight at 4 °C, secondary antibody incubated, DAB developed, hematoxylin re-stained, blocked, and images were acquired. Primary antibodies used were G6PC (rabbit, Abclonal, ab20193, 1:200, Woburn, MA), N-Cadherin (mouse, Raybiotech, 14461618, 1:100, Norcross, GA).

### Western blotting

Proteins were extracted from cells or mouse kidneys using RIPA lysis buffer (P0013B, Beyotime Biotech, Shanghai, China). Protein concentration was determined using a BCA protein assay, and samples were then dissolved in 5X SDS-PAGE loading buffer. Proteins were separated by 8% SDS-PAGE and electrotransferred onto a PVDF membrane (Merck Millipore, Darmstadt, Germany). The membrane was incubated in blocking buffer (5% skim milk, 20 mM Tris–HCl, 150 mM NaCl, pH 8.0, and 0.1% Tween 20) for 1 h at room temperature. Following blocking, the membrane was incubated at 4 °C overnight with primary antibodies against fibronectin (FN) (ab23750, Abclonal, Port Talbot, UK, 1:5000), Collagen I (SC293182, Santa, Dallas, TX, 1:5000), N-Cadherin (SC59987, Santa, Dallas, TX, 1:1000), phosphorylated Smad3 (pSmad3) (ET1609-41, HUABIO, Hangzhou, China, 1:1000), Vimentin (ET, 1610-39, HUABIO, Hangzhou, China, 1:5000), α-smooth muscle actin (α-SMA) (ET1607-53, HUABIO, Hangzhou, China, 1:1000), GAPDH (6004-1-1g, Proteintech, Shanghai, China, 1:5000), Snail (A11794, Abclonal, Woburn, MA, 1:1000), PCK1 (A2036, Abclonal, Port Talbot, UK, 1:1000), G6PC (A20193, Abclonal, Port Talbot, UK, 1:1000), and FBP1 (14405406, Raybiotech, 1:500, Norcross, GA).

After washing, binding of primary antibodies was detected using the ECL method (180-501 ECL, Tanon, Shanghai, China) with horseradish peroxidase-coupled secondary antibodies (goat anti-rabbit IgG, A0208 or goat anti-mouse IgG, A0216, Beyotime Biotech, Shanghai, China). Band density was measured using Quantity One software (Bio-Rad, Hercules, CA).

### Data analysis

Results are expressed as the mean ± standard deviation (*X* ± SD). Differences among multiple groups were analyzed using one-way ANOVA, and comparisons between two groups were performed with an unpaired Student’s *t*-test. Data analysis was conducted using SPSS 26.0 (SPSS Inc., Chicago, IL) and GraphPad Prism 8.0 software (GraphPad Software, La Jolla, CA). A *p* value <.05 was considered statistically significant.

## Results

### Tanshinone I ameliorates renal fibrosis and promotes gluconeogenesis in the UUO mouse model

**Figure 1.**
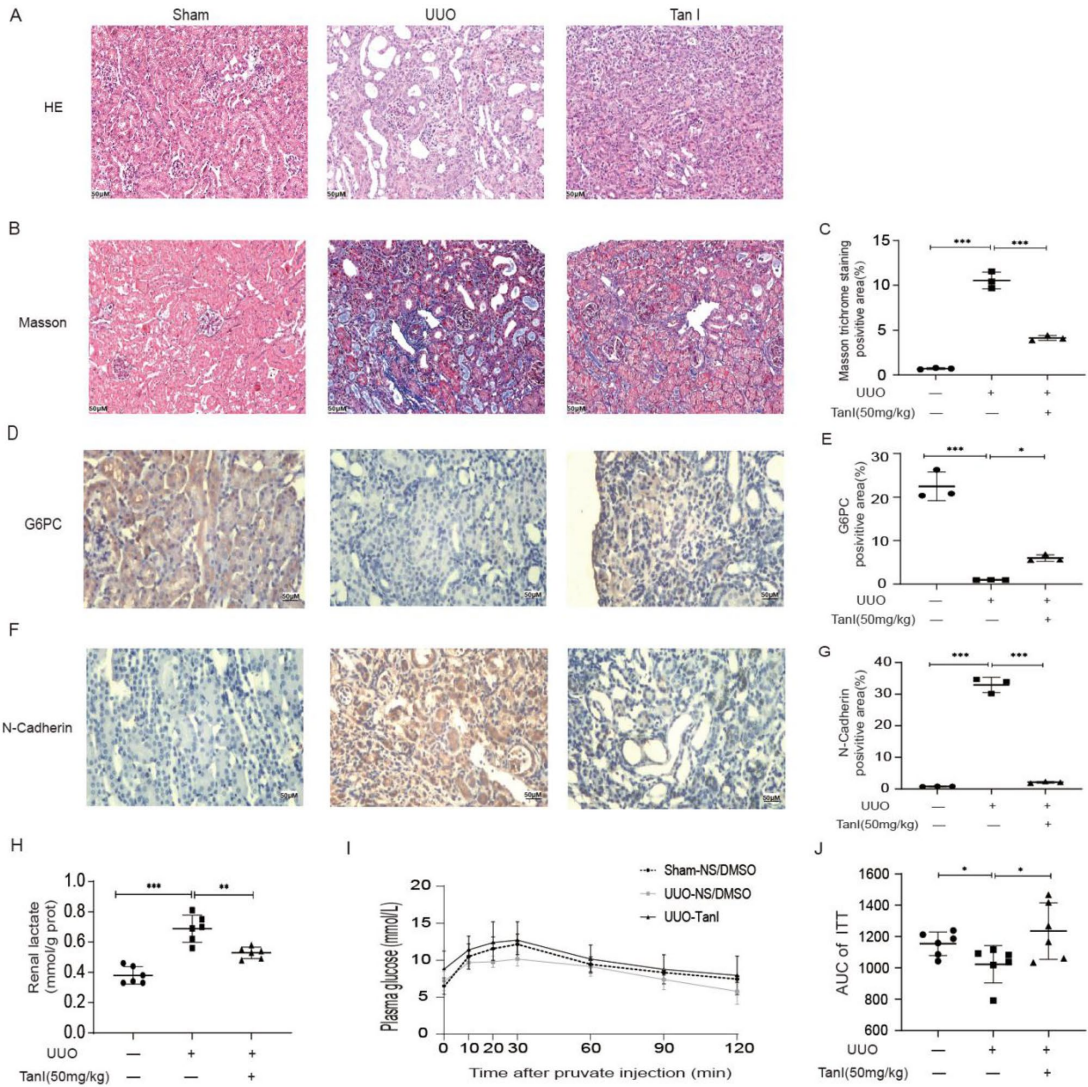
Tan-I mitigates renal pathological damage and improves gluconeogenesis in UUO mice. (A) Representative photomicrographs of H&E staining in renal tissues (original magnification ×400). (B, C) Representative photomicrographs of Masson staining and the IOD for collagen deposition (blue) in renal tissues (original magnification ×400). (D–G) Representative images of G6PC and N-Cadherin expression detected by IHC. Quantitative analysis of G6PC and N-Cadherin positive area accelerated renal lactate clearance following tanshinone I intervention in UUO mice. (H–J) Glucose tolerance test performed on the ninth day post-UUO operation with intraperitoneal injection of sodium pyruvate; blood glucose recovery levels were measured at different time points and statistically compared. *N* = 6 in each experimental group. Data are presented as mean ± SD. *p < .05, **p < .01, and ***p < .001.

**Figure 2.**
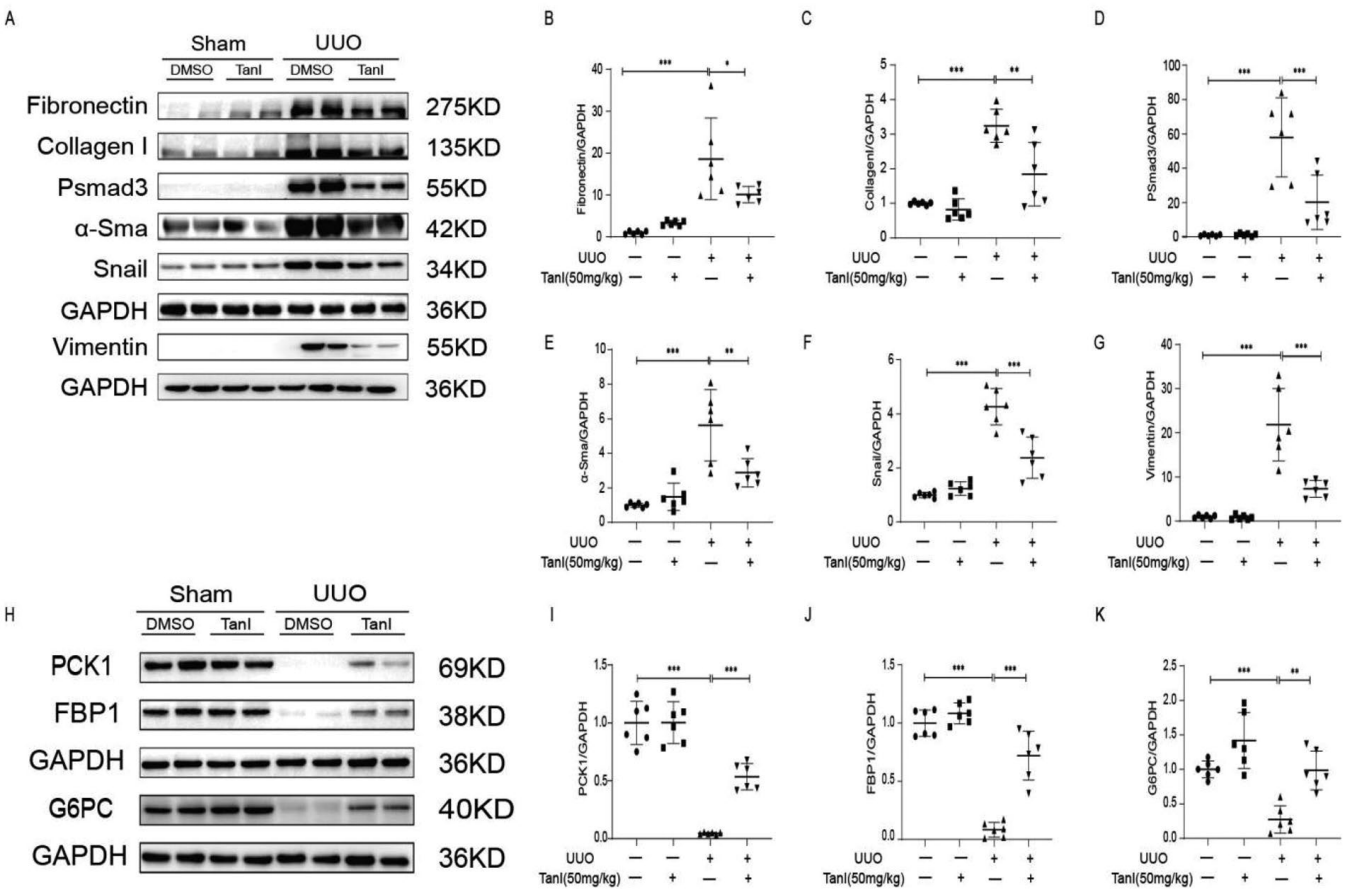
Tan-I mitigates renal fibrosis and restores gluconeogenesis in UUO mice. (A–G) Western blot analysis and quantification of FN, Collagen I, pSmad3, α-SMA, Snail, and Vimentin expressions. (H–K) Western blot analysis and quantification of PCK1, FBP1, and G6PC expressions. *N* = 6 in each experimental group; one representative of at least three independent experiments is shown. Data are presented as mean ± SD. *p < .05, **p < .01, and ***p < .001.

In the *in vivo* study, the UUO mouse model was used to evaluate the effect of Tan I on renal fibrosis. Histological analyses with HE and Masson staining revealed that the UUO mouse model exhibited renal interstitial fibrosis, which was alleviated following Tan I treatment (Figure 1(A–C)). Immunohistochemical results showed that G6PC was predominantly distributed in renal tubular cells and its expression was reduced in UUO and increased after Tan I intervention, and N-Cadherin, a marker of EMT expression, was elevated in UUO and its expression was reduced by Tan I (Figure 1(D–G). Additionally, Tan I significantly reduced lactate accumulation in the kidneys of UUO mice (Figure 1(H)). Glucose tolerance assays indicated that sodium pyruvate levels were decreased in the kidneys of UUO mice, but Tan I treatment restored these levels (Figure 1(I,J)). The expression of fibrosis marker proteins, including FN, N-Cadherin, pSmad3, α-SMA, Vimentin, and Snail, was significantly elevated in the kidneys of UUO mice compared to sham-operated controls. In contrast, the expression of these proteins was markedly reduced in the Tan I-treated group (Figure 2(A–G)). Furthermore, gluconeogenesis-related proteins FBP1, G6PC, and PCK1 were downregulated in the kidneys of UUO mice, and Tan I treatment reversed these changes (Figure 2(H–K)). Collectively, these findings suggest that Tan I not only alleviates renal fibrosis but also protects against fibrosis by promoting gluconeogenesis, regulating renal metabolism, decreasing lactate accumulation, and restoring sodium pyruvate levels.

### HK2 cells: tanshinone I ameliorates fibrosis associated with gluconeogenesis

**Figure 3.**
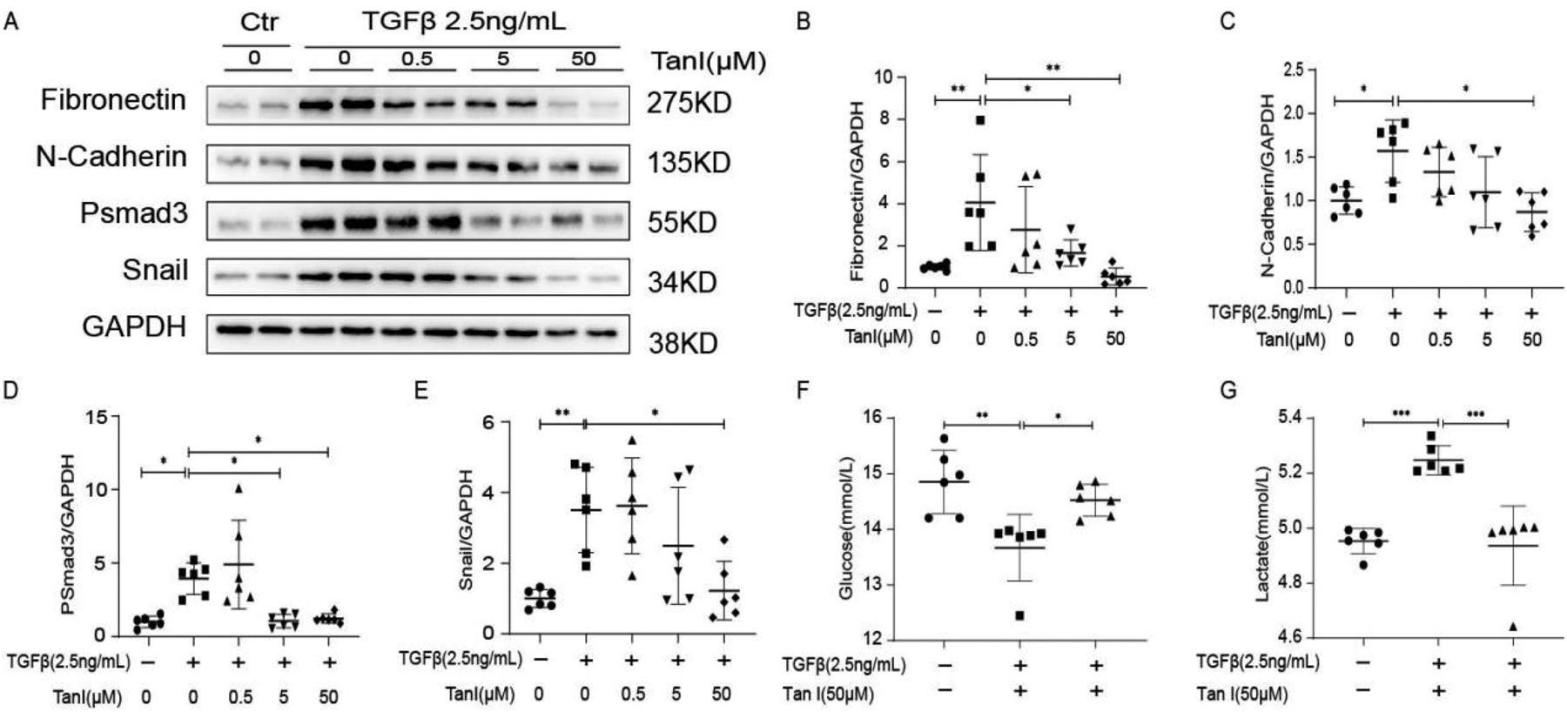
Tanshinone I (Tan-I) attenuates fibrotic changes and affects cellular metabolism in human renal cortical proximal tubule (HK2) cells. HK2 cells were starved for 8 h and then treated with various concentrations (0.5 μM, 5 μM, and 50 μM) of Tan-I for 48 h. (A–E) Western blot analysis and quantification of FN, N-Cadherin, pSmad3, α-SMA, and Snail expressions. (F–G) Metabolic levels of glucose and lactate in cell supernatants at 48 h were measured and quantified. Data are presented as mean ± SD. *p < .05, **p < .01, and ***p < .001. *N* = 6 in each experimental group; one representative result from at least three independent experiments is shown.

We induced fibrosis in HK2 cells using 2.5 ng/mL of TGFβ to validate the antifibrotic effect of Tan I. Results showed that Tan I effectively ameliorated fibrosis in HK2 cells. The safe concentration of Tan I was determined according to its drug insert and related literature [24]. Three concentrations 0.5 μM, 5 μM, and 50 μM were used, with cells treated for 48 h. Western blotting results revealed that Tan I significantly reduced the expression of FN, N-Cadherin, pSmad3, and Snail (Figure 3(A–E)). Additionally, the 50 μM concentration of Tan I significantly affected glucose and lactate levels in HK2 cell culture supernatants after 48 h of treatment. Tanshinone I reduced lactate accumulation while increasing glucose levels (Figure 3(F,G)). These results suggest that Tan I not only exerts an antifibrotic effect by inhibiting fibrosis-related protein expression but also affects cellular metabolism by reducing lactate accumulation and elevating glucose levels.

### Mouse primary renal tubular cells: tanshinone I ameliorates fibrosis by promoting gluconeogenesis

**Figure 4.**
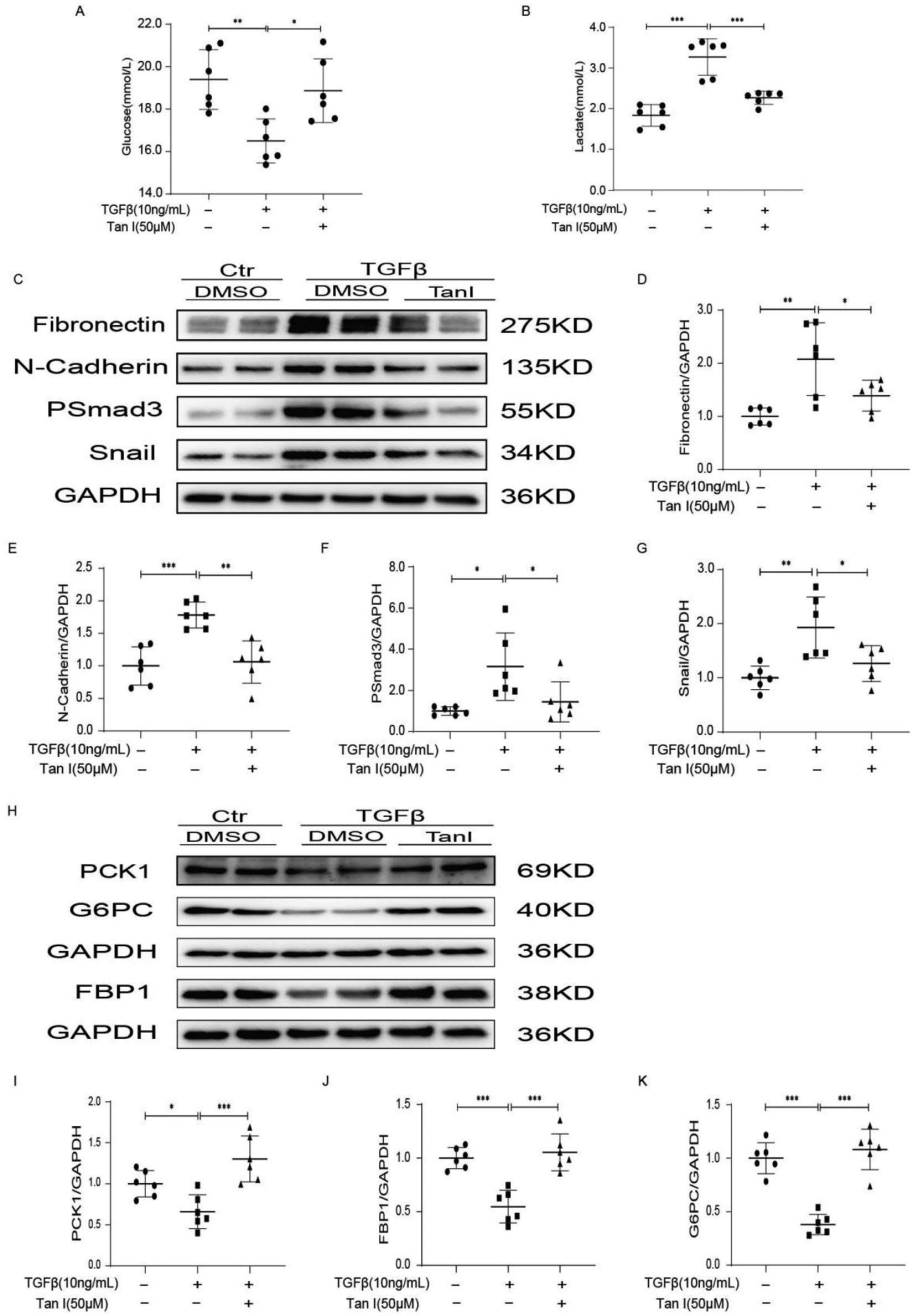
Tan-I ameliorates renal fibrosis, restores gluconeogenesis, and improves cellular metabolism in mouse primary renal tubular cells. (A, B) Metabolic levels of glucose and lactate in cell supernatants at 48 h were measured and quantified. (C–G) Western blot analysis and quantification of FN, N-Cadherin, pSmad3, and Snail expressions. (H–K) Western blot analysis and quantification of PCK1, FBP1, and G6PC expressions. Data are presented as mean ± SD. *p < .05, **p < .01, and ***p < .001. *N* = 6 in each experimental group; one representative result from at least three independent experiments is shown.

The effect of Tan I on antifibrosis was investigated in mouse primary renal tubular cells. Primary renal tubular cells were induced with 10 ng/mL of TGFβ to establish a fibrosis model. Subsequently, 50 μM Tan I was used for intervention, and cell supernatants and protein samples were collected after 48 h. Analysis of the supernatant showed that lactate levels decreased and glucose levels increased in the Tan I-treated group (Figure 4(A,B)). Experimental results indicated that Tan I inhibited renal fibrosis (Figure 4(C–G)) and restored the expression of gluconeogenesis-related proteins FBP1, PCK1, and G6PC compared to the model group (Figure 4(H–K)). These findings suggest that Tan I significantly ameliorated TGFβ-induced renal tubular cell fibrosis by promoting gluconeogenesis.

### Tanshinone I ameliorates renal fibrosis by promoting gluconeogenesis through up-regulation of PGC1α

**Figure 5.**
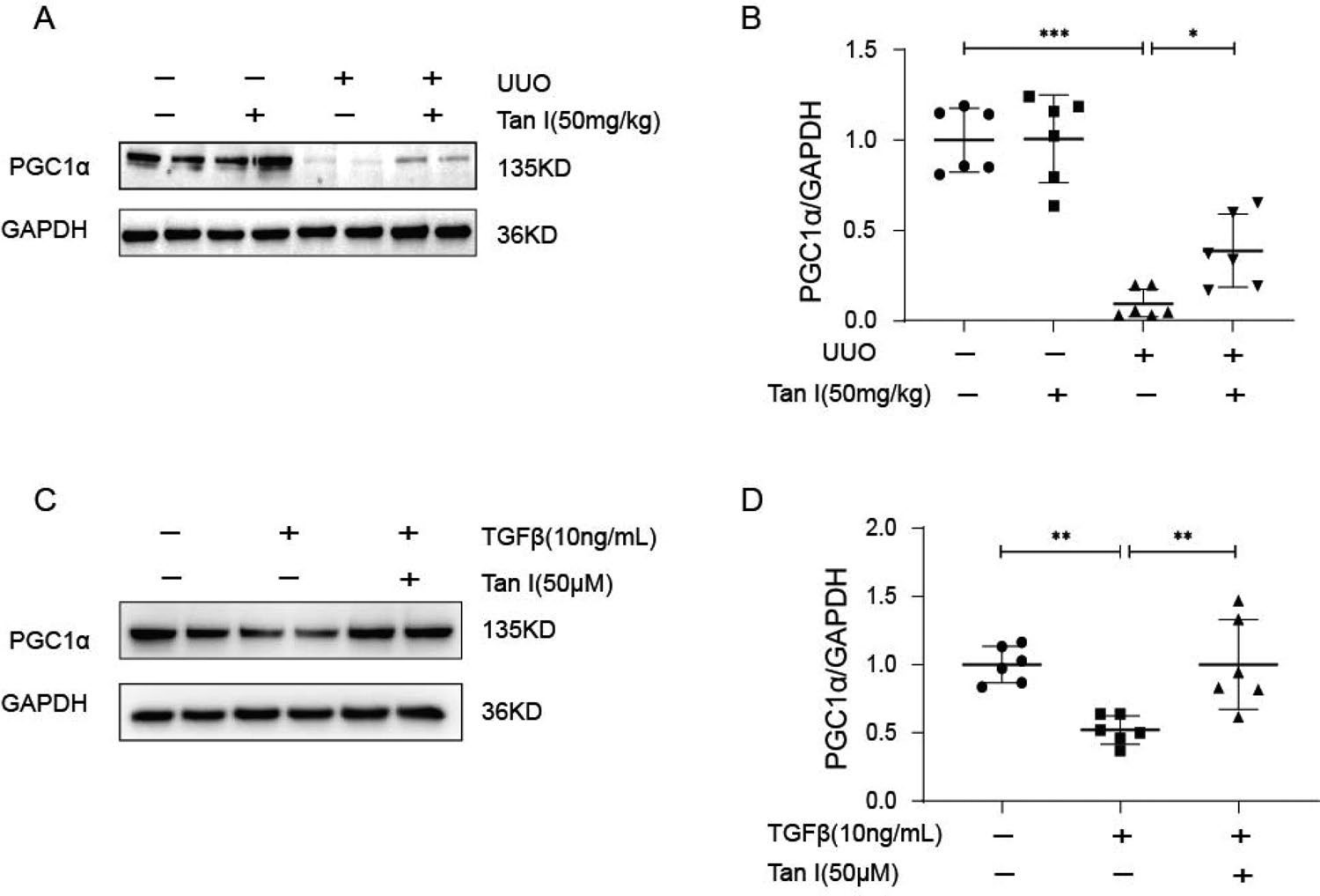
Tan-I upregulates PGC1α expression *in vitro* and *in vivo*. (A, B) Western blot analysis and quantification of PGC1α expression in sham and UUO kidneys. (C, D) Primary renal tubular cells were starved for 8 h and then treated with 50 μM Tan-I for 48 h. Western blot analysis and quantification of PGC1α expression were performed. Data are presented as mean ± SD. *p < .05, **p < .01, and ***p < .001. *N* = 6 in each experimental group; one representative result from at least three independent experiments is shown.

In the UUO mouse model, PGC1α expression was reduced but significantly elevated following Tan I intervention. Similarly, in primary renal tubular cells, TGFβ stimulation significantly reduced PGC1α expression, which was restored by Tan I treatment (Figure 5(A–D)). These results suggest that Tan I ameliorates renal fibrosis by regulating PGC1α expression and promoting gluconeogenesis.

### The PGC1α inhibitor suppresses the effect of tanshinone I in improving renal fibrosis

**Figure 6.**
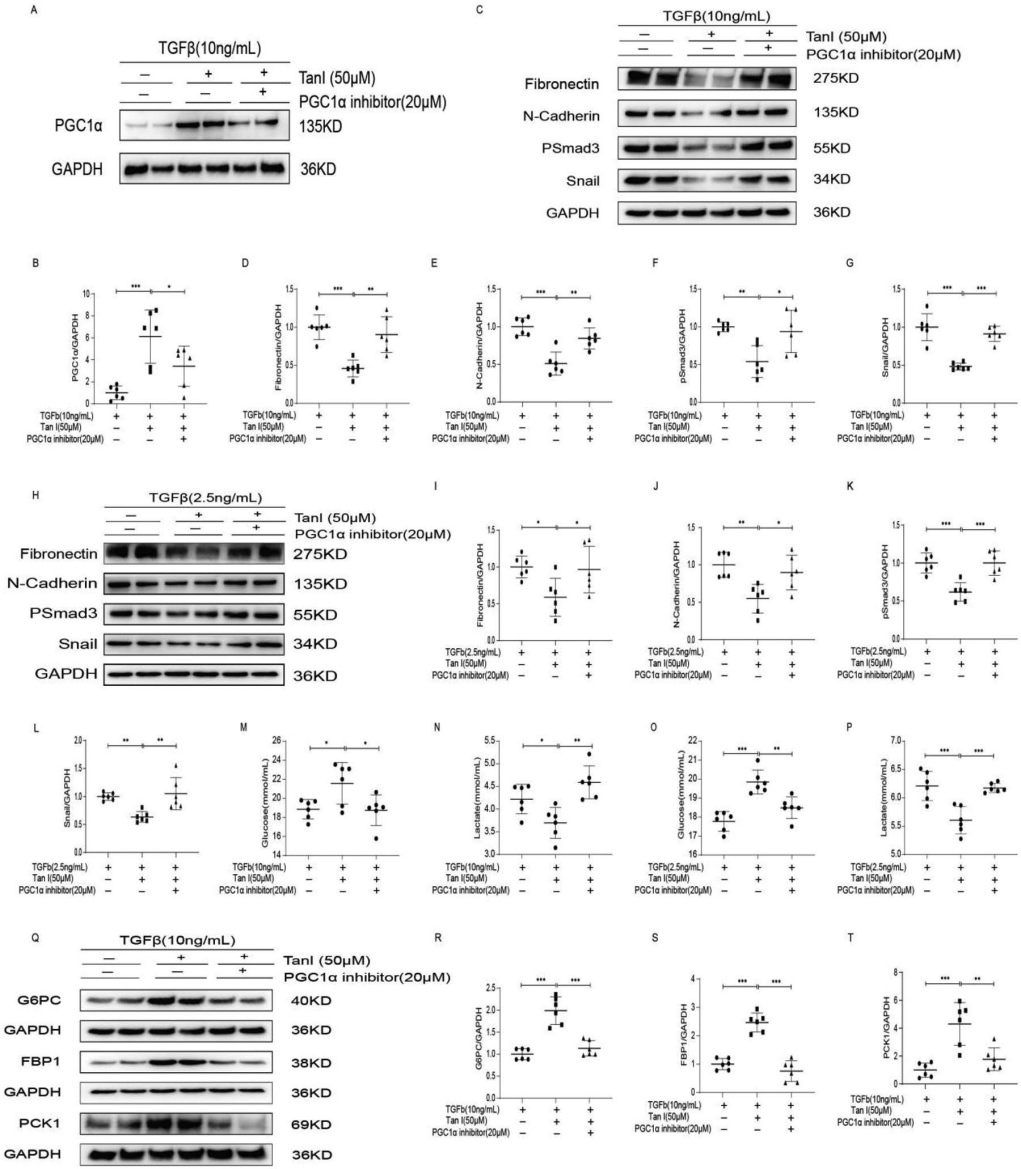
Effects of Tan-I on fibrosis and gluconeogenesis are inhibited by PGC1α inhibitors. (A, B) Western blot analysis and quantification of PGC1α expression in primary renal tubular cells following administration of the PGC1α inhibitor. (C–L) Western blotting results showing that the antifibrotic effects of Tan-I are reversed by the PGC1α inhibitor in both primary renal tubular cells and HK2 cells. (M–P) Measurement of glucose and lactate levels in cell supernatants, revealing that the PGC1α inhibitor mitigates Tan-I’s effects on cellular metabolism in primary renal tubular cells and HK2 cells. (Q–T) Western blotting results showing that the restoration of gluconeogenesis by Tan-I is reversed by PGC1α inhibitors. Data are presented as mean ± SD. *p < .05, **p < .01, and ***p < .001. *N* = 6 in each experimental group; one representative result from at least three independent experiments is shown.

In the fibrosis model using TGFβ-stimulated primary renal tubular cells, WB analysis showed that PGC1α expression was suppressed by the PGC1α inhibitor (Figure 6(A,B)). The therapeutic effect of Tan I on fibrosis was also diminished by the PGC1α inhibitor in both HK2 and primary renal tubular cells (Figure 6(C–L)). Additionally, PGC1α inhibitors reduced Tan I’s efficacy in alleviating lactate accumulation and restoring glucose, as evidenced by cell supernatant assays (Figure 6(M–P)). Western blot analysis further revealed that the ability of Tan I to restore gluconeogenesis was impaired by PGC1α inhibitors (Figure 6(Q–T)). These findings underscore the pivotal role of PGC1α in mediating the effects of Tan I on renal fibrosis and gluconeogenesis.

## Discussion

A growing body of research indicates that both structural and functional damage from fibrosis inhibits gluconeogenesis, and reduced gluconeogenesis exacerbates fibrosis progression through various mechanisms, creating a vicious cycle that is particularly pronounced in patients with CKD [25,26]. In healthy individuals, the kidney plays a pivotal role in maintaining glucose homeostasis through both gluconeogenesis and glucose reabsorption. In patients with CKD, renal fibrosis results in the structural disruption of the tubulo-interstitium, impairing the function of proximal tubular cells. This impairment in tubular reabsorption and gluconeogenic activity reduces the kidney’s ability to maintain normoglycemia, further aggravating glucose metabolism disorders [6,27,28]. Inflammation and oxidative stress associated with fibrosis alter the metabolic environment of the kidney, inhibiting the expression and activity of gluconeogenic enzymes. Transforming growth factor-beta (TGF-β) acts as a pro-fibrotic factor that not only promotes fibrosis but also impairs gluconeogenesis by downregulating enzymes such as PCK1, FBP1, and G6PC [29,30]. During fibrosis, the energy metabolism of renal tubular cells is disrupted, and mitochondrial dysfunction results in diminished ATP production. This state of energy deficiency further suppresses the activity of the gluconeogenic pathway [31]. Reduced gluconeogenesis leads to an inadequate local energy supply in the kidney, affecting cellular repair and regenerative capacity, thereby exacerbating fibrosis progression [25]. Both lactate accumulation and reduced gluconeogenesis are linked with worsening fibrosis [32,33]. Lactate accumulation due to reduced gluconeogenesis causes acidification of the intracellular environment and activates pro-fibrotic gene expression. For instance, lactate can enhance the expression of fibrosis-related genes by activating the reactive oxygen species (ROS) and TGF-β signaling pathways [32]. Conversely, the restoration of gluconeogenesis helps sustain normal cellular metabolic functions [34,35]. Tanshinone I improves cellular metabolic status by reducing lactate levels and increasing gluconeogenesis, which is crucial for mitigating fibrosis. In the UUO model, reduced renal gluconeogenesis is observed as an early response in renal tubular cells [30,36]. We observed a significant decrease in the expression of gluconeogenic enzymes in both the UUO model and TGF-β-stimulated primary renal tubular cell experiments, which was reversed following Tan I treatment. These findings suggest that targeting renal gluconeogenesis could be a promising approach for fibrosis treatment.

Studies have demonstrated that PGC1α is an upstream regulator of several key enzymes involved in gluconeogenesis. It promotes glucose production by modulating the expression of enzymes such as PCK1, G6PC, and FBP1, which are essential for maintaining blood glucose levels and energy homeostasis [14,37]. PGC1α is highly expressed in the kidneys and plays a protective role. When renal gluconeogenesis is impaired, PGC1α expression is also reduced [37]. PGC1α prevents fibrosis formation by regulating fibroblast activity and inhibiting excessive proliferation and collagen over-synthesis [38,39]. Additionally, PGC1α slows fibrosis progression by enhancing the expression of antioxidant enzymes, thereby reducing cellular damage from oxidative stress [40–42]. It also regulates inflammation-related gene expression, minimizing tissue damage caused by chronic inflammation [38,43,44]. PGC1α integrates various metabolic and stress signaling pathways, including AMPK and SIRT1, which work synergistically to regulate cellular metabolism, antioxidant defenses, and antifibrotic processes [40,45]. By promoting gluconeogenesis and providing the energy required for cellular repair and normal function, PGC1α mitigates fibrosis progression [46–48]. PGC1α is crucial for maintaining normal cellular metabolic status and function. Compared to normal controls, PGC1α transcription is inhibited in CKD patients, nephropathy fibrosis models, and TGFβ-stimulated *in vitro* models [30]. However, renal fibrosis can be attenuated by using PGC1α agonists [17]. Our experiments further validated this finding, showing that PGC1α expression was significantly reduced under TGFβ stimulation, leading to inhibited gluconeogenesis. Tanshinone I significantly upregulated PGC1α expression and restored the levels of gluconeogenesis-related enzymes such as PCK1, FBP1, and G6PC. This suggests that PGC1α plays a crucial role in the anti-fibrotic effects of Tan I through the gluconeogenesis pathway. The promotion of gluconeogenesis and the antifibrotic effects of Tan I were significantly attenuated by the addition of a PGC1α inhibitor, further confirming the indispensable role of PGC1α in this process.

Tanshinone I has been demonstrated to ameliorate renal fibrosis in our previous experiments; however, its precise mechanism of action remains unclear. Building on this research, we investigated how Tan I improves renal fibrosis. Initially, WB experiments confirmed that TGFβ stimulation increased the expression of fibrotic proteins such as FN, N-Cadherin, pSmad3, α-SMA, and Snail in HK2 cells, while Tan I treatment reduced their expression. Concurrently, TGFβ stimulation impaired lactate clearance and decreased glucose production in cell supernatants. In contrast, Tan I mitigated lactate accumulation and enhanced glucose production. These results suggest that Tan I ameliorates fibrosis while modulating glucose and lactate metabolism. Subsequently, in *in vivo* experiments, we observed reduced gluconeogenesis expression in the fibrosis model. Tanshinone I restored renal gluconeogenesis, reduced lactate accumulation, and improved renal fibrosis. These findings were further validated in primary renal tubular cells, where Tan I accelerated lactate clearance, increased glucose levels, restored gluconeogenesis, and reduced renal fibrosis. Additionally, we noted decreased PGC1α expression in the kidneys of UUO model mice and TGFβ-stimulated primary renal tubular cells, which was elevated following Tan I treatment. The beneficial effects of Tan I on renal fibrosis and gluconeogenesis were diminished when PGC1α expression was inhibited. These results suggest that Tan I improves renal fibrosis by upregulating PGC1α, which promotes gluconeogenesis.

Numerous studies have highlighted the crucial role of PGC1α in nephroprotection; however, few have investigated how upregulating PGC1α to promote gluconeogenesis can ameliorate renal fibrosis. This study is the first to elucidate the pathway by which Tan I exerts its effects, thereby enriching our understanding of PGC1α’s regulatory mechanisms in renal diseases and suggesting new therapeutic avenues for Tan I in renal fibrosis treatment. As a natural pharmaceutical agent, Tan I exhibits a favorable safety profile and multiple pharmacological effects. This research explores its potential therapeutic benefits in renal fibrosis, providing a theoretical foundation for its clinical application. Despite these important findings, the study has certain limitations, including reliance on a single animal model and an *in vitro* cell model, and the lack of clinical data. Future studies should include transgenic animal models and clinical trials to further validate the mechanism of action and therapeutic efficacy of Tan I.

## Conclusions

This study reveals, for the first time, the specific mechanism by which Tan I ameliorates renal fibrosis by promoting gluconeogenesis through upregulation of PGC1α. Our findings provide new insights into the metabolic therapy of renal fibrosis and have significant clinical implications. Future research will aim to validate these findings and support the clinical application of Tan I and its analogs.

## Data Availability

The datasets used and/or analyzed during the current study are available from the corresponding author on reasonable request.
